# Vesicocutaneous Fistula Presenting Groin Abscess and Chronic Osteomyelitis in Pubic Bone

**DOI:** 10.4055/cios.2009.1.3.176

**Published:** 2009-08-17

**Authors:** Sang Bum Kim, Woong Kyo Jung, Dong Ik Song, Soon Hyuck Lee

**Affiliations:** Department of Orthopedic Surgery, Korea University Anam Hospital, Korea University College of Medicine, Seoul, Korea.

**Keywords:** Bladder, Fistula, Chronic osteomyelitis

## Abstract

The authors report a case of bladder fistula associated with a medial thigh cutaneous fistula and chronic osteomyelitis of the pubic bone 11 years after surgery for a pelvic bone fracture and bladder rupture. In the presenting case, despite the clinical suspicion, none of the diagnostic tools demonstrated the bladder fistula preoperatively. This case suggests that bladder repair should be prepared, even if the bladder fistula cannot be confirmed by imaging studies because the amount of urine leakage can be minimal or the fistula can close spontaneously.

Disruptions of the pelvic ring are associated with a 7% to 25% incidence of lower urinary tract injuries.1) In particular, the prevalence of bladder rupture associated with a pelvic fracture has been reported to be 5% to 10%. On the other hand, the incidence of pelvic fractures exceeds 80% in patients with bladder ruptures.2)

A bladder fistula is a rare complication after primary repair of a bladder rupture.3) We present a case of a groin abscess associated with chronic osteomyelitis in the pubic bone caused by a vesicocutaneous fistula, 11 years after surgery to treat a pelvic fracture and bladder rupture.

In the presenting case, the vesicocutaneous fistula was suspected clinically but the bladder fistula could not be observed preoperatively using any of the diagnostic tools. The suspected bladder fistula was confirmed only by a vigorous search during surgical exploration.

## CASE REPORT

A 57-year-old man was admitted to our department for erythematous swelling of his left groin, which had become symptomatic one month earlier.

Eleven years ago, the patient suffered a pelvic bone fracture along with a bladder rupture as a result of an automobile accident. He underwent surgical treatment at another hospital. Seven years ago, the patient had suffered a cutaneous sinus at the pubic area associated with pubic bone osteomyelitis, and underwent drainage and the removal of hardware at the same hospital.

**Fig. 1:**
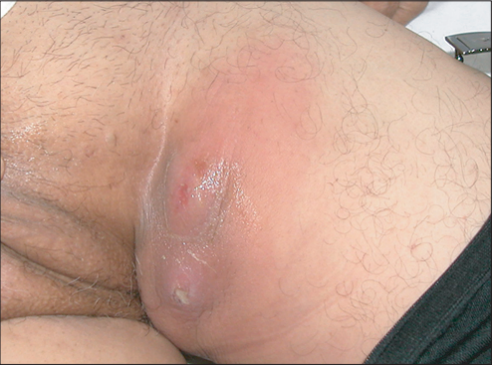
Clinical photograph: The left groin was swollen with erythematous change and a small fistulous opening was found at the center of the lesion.

This year, he reported symptoms of a urinary tract in fection repeatedly over a period of several months, which was not resolved by oral antibiotics. When transferred to our institution, he was febrile and the left groin showed erythematous swelling with a small fistulous opening, which secreted a serous discharge with a urine odor (Fig. 1).

**Fig. 2:**
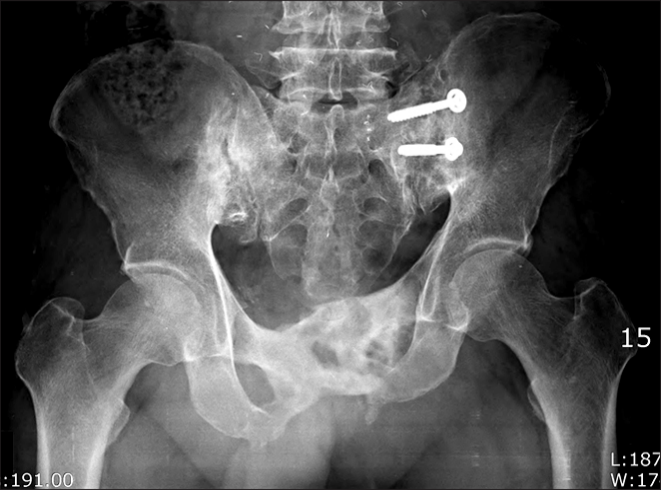
Initial anteroposterior radiograph of the pelvis.

**Fig. 3:**
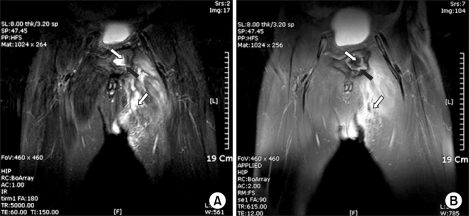
MRI shows an abscess (white arrow) within the deformed pubic ramus and the adductor longus, extending to the subcutaneous tissue, and osteomyelitis (black arrow) in the adjacent bone.

**Fig. 4:**
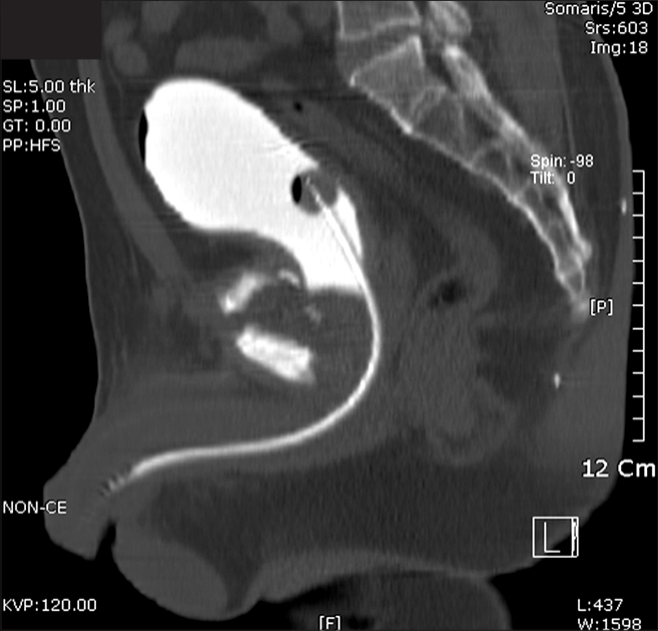
CT cystogram shows no contrast connections from the bladder to the abscess of the pubis and thigh.

The radiographs showed osteolytic and sclerotic bony lesions in the pubic ramus, suggesting bony sequestrum (Fig. 2). Magnetic resonance imaging (MRI) suggested an abscess within the pubic ramus and adductor longus extending to the subcutaneous tissue, as well as osteomyelitis in the adjacent bone (Fig. 3). A cystography showed no communication between the bladder and abscess. A computed tomography (CT) cystogram revealed an abscess containing air densities within the bony defect of the deformed pubic ramus. There were no contrast connections from the bladder to the abscess of the pubis and thigh (Fig. 4).

**Fig. 5:**
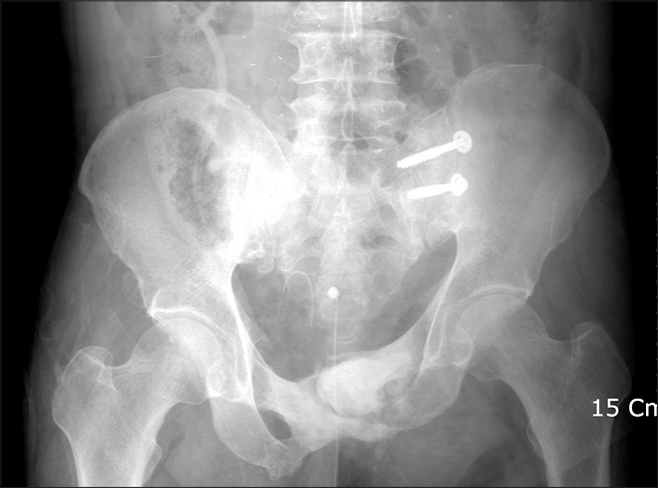
Postoperative anteroposterior radiograph of the pelvis.

The fistulous tract from the groin abscess to pubic ramus was excised. The tract was obstructed at the midway. Thorough debridement of the sequestrum and fibrous infected tissue in the cavity of the pubic bone was performed after making a transverse skin incision just over the symphysis pubis. Subsequently, a search was made to obtain proof of leakage from the bladder. The tiny amount of clear fluid was found to leak from the fibrous tissue in the destructive cavity of the pubic bone adjacent to the bladder. It merged instantly with oozing blood and was difficult to differentiate from soft tissue bleeding. The bladder was filled with indigo carmine through a Foley catheter to determine if the fluid was urinary leakage. Communication of the pubic ramus with the bladder was verified. A further dissection around the hole was performed to identify the location of the hole precisely. The pubic cortex and bladder wall adhered to each other and the bladder fistula was found behind the hole of the cortex and connected directly into the destructive cavity of the pubic bone. Antibiotics-mixed bone cement was packed into the pubic bone to eradicate the osteomyelitis and fill the bone defect (Fig. 5). The bladder fistula and surrounding inflammatory tissue were excised and repaired in two layers, and an omentoplasty was performed by a urologic surgeon.

The urine and tissue cultures confirmed Pseudomonas aeruginosa. Appropriate antibiotics were administered after reporting the culture results. The Foley catheter was removed on postoperative day 13 and normal voiding was restored. After surgery, recovery was uneventful without a recurrence of the infection or the bladder fistula at the 1 year follow-up.

## DISCUSSION

The common causes of vesicocutaneous fistula include extensive trauma with pelvic fractures, after irradiation for pelvic malignancies and postoperative causes, such as radical hysterectomy and hip arthroplasty.4) Fistulography, cystography and cystoscopy can help in diagnosing vesicocutaneous fistula. Cross-sectional techniques, such as CT scan and MRI, are becoming increasingly useful for diagnosis, and are considered to be the primary test in some cases.^5^-7)

In this case, the authors suspected a vesicocutaneous fistula clinically due to the serous urine-like discharge secreted from the erythematous swelling in his groin, and the patient had a recurrent history of a urinary tract infection. Despite the clinical suspicion, cystography, CT cystography and MRI could not demonstrate the bladder fistula. The clinical suspicion of bladder leakage led to a vigorous search for the leakage during surgery. Intraoperatively, the small amount of urinary leakage was not definite because the oozing blood from the adjacent soft tissue obscured the urinary leakage. A confirmative study using indigo carmine confirmed that the bladder fistula was connected to the destructive pubic bone. A failure of several imaging studies to demonstrate the leakage appears to be because the bladder fistula had closed spontaneously before surgery. It should be emphasized that the failure to demonstrate a bladder fistula by imaging studies does not exclude its presence in a clinically suspicious situation.

The authors report a vesicocutaneous fistula associated with medial thigh cutaneous fistula and chronic osteomyelitis of the pubic bone 11 years after undergoing surgery for a pelvic bone fracture and bladder rupture. This case suggests that bladder repair should be prepared, even if bladder fistula cannot be confirmed by imaging studies in a clinically suspected case because the amount of urine leakage may be minimal or the fistula could closed spontaneously.
